# Long-term effects of bisphosphonate therapy: perforations, microcracks and mechanical properties

**DOI:** 10.1038/srep43399

**Published:** 2017-03-06

**Authors:** Shaocheng Ma, En Lin Goh, Andi Jin, Rajarshi Bhattacharya, Oliver R. Boughton, Bhavi Patel, Angelo Karunaratne, Nghia T. Vo, Robert Atwood, Justin P. Cobb, Ulrich Hansen, Richard L. Abel

**Affiliations:** 1 Department of Mechanical Engineering, Faculty of Engineering, Imperial College London, London, SW7 2AZ, United Kingdom; 2 MSk Laboratory, Department of Surgery and Cancer, Faculty of Medicine, Imperial College London, London, W6 8PR, United Kingdom; 3 St. Mary’s Hospital, North West London Major Trauma Centre, Imperial College, London, W2 1NY, United Kingdom; 4 Department of Mechanical Engineering, Faculty of Engineering, University of Moratuwa, Moratuwa, 10400, Sri Lanka; 5 Diamond Light Source Ltd, Harwell Science and Innovation Campus, Didcot, OX11 0DE, United Kingdom

## Abstract

Osteoporosis is characterised by trabecular bone loss resulting from increased osteoclast activation and unbalanced coupling between resorption and formation, which induces a thinning of trabeculae and trabecular perforations. Bisphosphonates are the frontline therapy for osteoporosis, which act by reducing bone remodelling, and are thought to prevent perforations and maintain microstructure. However, bisphosphonates may oversuppress remodelling resulting in accumulation of microcracks. This paper aims to investigate the effect of bisphosphonate treatment on microstructure and mechanical strength. Assessment of microdamage within the trabecular bone core was performed using synchrotron X-ray micro-CT linked to image analysis software. Bone from bisphosphonate-treated fracture patients exhibited fewer perforations but more numerous and larger microcracks than both fracture and non-fracture controls. Furthermore, bisphosphonate-treated bone demonstrated reduced tensile strength and Young’s Modulus. These findings suggest that bisphosphonate therapy is effective at reducing perforations but may also cause microcrack accumulation, leading to a loss of microstructural integrity and consequently, reduced mechanical strength.

Osteoporosis is a metabolic bone disorder affecting 200 million people worldwide, with a high prevalence in the ageing population^1^. Osteoporosis increases the risk of fragility fracture^2^,^3^,^4^,^5^,^6^, and is estimated to contribute to 8.9 million fractures annually^7^. Fractures, which commonly occur at the hip and spine^8^,^9^ result in disability, mortality and high healthcare costs, thereby placing a huge strain on the healthcare system^10^. The treatment of osteoporosis costs nearly $17 billion per year in the United States^11^ and €37 billion per year in the European Union^12^. With the rise in life expectancy, the incidence and cost of fragility fractures are projected to increase even further in the future.

Osteoporosis is characterised by a loss of bone mass and can be defined clinically using dual X-ray absorptiometry (DXA) as a T-score less than 2.5 standard deviations below the mean bone mineral density (BMD) in young adults^13^,^14^. Although osteoporosis causes bone to become fragile, studies have shown a poor correlation between osteoporosis, as defined by DXA, and the occurrence of fractures in the ageing population^15^,^16^. It is evident that more than half of these individuals who suffer from fractures do not meet the clinical diagnosis of osteoporosis^17^. These findings have led to the emergence of the concept of bone quality, which is now widely accepted as a key element in understanding bone fragility, in addition to bone mass^18^,^19^. Bone quality refers to the material and structural properties of bone that make it resistant to fracture including bone turnover, collagen and mineral matrix, microarchitecture and accumulated microdamage^6^. These factors are all thought to play a significant role in the development of fragility fractures^20^,^21^,^22^,^23^ and are therefore relevant for understanding the efficacy of treatment.

Bisphosphonates such as alendronate, risedronate and zoledronate are potent antiresorptive agents that form the first-line pharmacotherapy for the treatment of osteoporosis, with over 190 million prescriptions issued each year^24^,^25^. In osteoporosis, there is increased osteoclast activity leading to excessive thinning of trabeculae and the formation of perforations, which contributes to increased bone fragility^26^. Trabecular perforation is caused by osteoclasts resorbing a cavity so deep that it cannot be refilled by osteoblasts^27^. Consequently, there is loss of trabecular connectivity which is one of the key determinants of the mechanical strength of bone^28^. Bisphosphonates are known to inhibit osteoclasts and increase BMD, thereby conferring a reduction in fracture risk^29^,^30^,^31^,^32^. It has also been suggested that bisphosphonate-inhibition of osteoclasts will restrict the formation of perforations^33^. Studies have shown bisphosphonates to be capable of reducing bone turnover by up to 90%, which persists throughout the duration of treatment and may reduce the risk of hip fractures by between 30–50%^34^,^35^,^36^.

Over the past decade, concerns have been raised by clinicians and researchers regarding the oversuppresion of remodelling caused by long-term bisphosphonate therapy, which may predispose the patient to fractures^37^,^38^,^39^,^40^,^41^,^42^. Indeed, there have been reports of spontaneous non-vertebral fractures associated with a substantial reduction in bone turnover in patients on long-term bisphosphonate therapy^43^. It has been suggested that bisphosphonate treatment causes an oversuppression of remodeling, resulting in the accumulation of microdamage, which compromises the mechanical properties of bone^43^,^44^. This is evident in both animal and human studies, where concurrent bisphosphonate therapy is associated with microdamage^45^,^46^,^47^. Research on the micro- and nano-structure of bone have reported an inverse relationship between the amount of microdamage and the mechanical strength^48^,^49^,^50^. Recently, Zimmermann *et al*. found bisphosphonate-treated bone to have reduced tissue strength compared to healthy bone^6^. However, no studies in humans have directly correlated the microstructural changes following bisphosphonate therapy with the mechanical properties. Thus, the present study aims to investigate the effect of bisphosphonate therapy on trabecular microstructure, including perforations and microcracks, and correlate this with the mechanical strength of bone.

## Materials and Methods

### Sample preparation

Trabecular bone samples were harvested from the femoral heads of three individual cohorts: a bisphosphonate-treated fracture group, an untreated fracture control group and a healthy ageing non-fracture control group. The fracture group samples were obtained from patients who underwent hip arthroplasty surgery for femoral neck fractures at Imperial College Healthcare National Health Service Trust in London, United Kingdom, while the non-fracture group samples were acquired from cadavers. All individuals with a history of primary bone disease or an underlying disorder such as cancer, which could lead to secondary bone disease were excluded from this study. The Imperial College Tissue Bank (R13004) granted ethical approval for the study and patients consented to the use of their tissue for research. All procedures performed in studies involving human participants were in accordance with the ethical standards of the institutional and/or national research committee and with the 1964 Helsinki Declaration and its later amendments or comparable ethical standards. A total of 21 samples were attained; eight from bisphosphonate-treated fracture patients (seven females; one male) (Table 1), eight from untreated fracture patients (seven females; one male) and five from cadavers of healthy ageing non-fracture individuals (four females; one male). The mean age of individuals was 79.3 ± 6.4 years in the bisphosphonate-treated group, 77.8 ± 3.4 years in the untreated fracture group and 77.8 ± 4.9 years in the healthy ageing non-fracture group. There was no significant difference present between the groups (One-Way ANOVA F = 0.5236, *p* = 0.644). Twenty-one cylindrical cores, 10 mm in height and 7 mm in diameter were taken from the region directly superior to the trabecular chiasma in the primary compressive trabecular arcade of the femoral heads. The cores were stored at −80 °C and only removed from storage for testing.

### Synchrotron X-ray micro-CT Experiment

**Figure 1:**
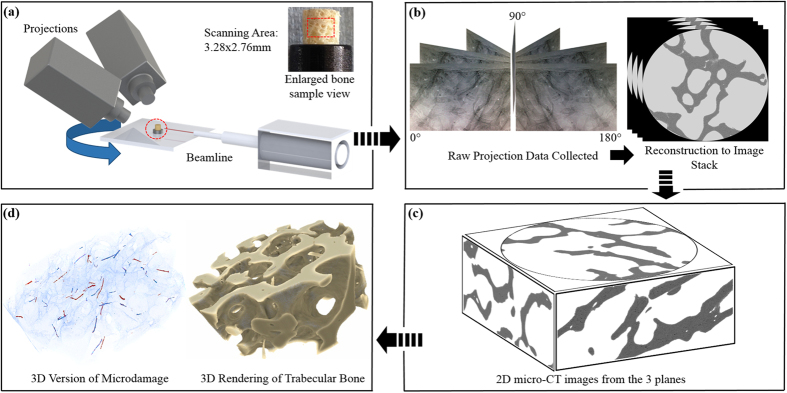
Synchrotron X-ray Micro-CT imaging of bone microstructure. (**a**) Bone cores were mounted in an X-ray beam line and rotated through 180°. A volume of interest (3.28 × 3.28 × 2.76 mm) was scanned at the centre of the core. (**b**) 640 X-Ray projections were collected at angular intervals of 0.28° and the projections were used to reconstruct 2000 axial slices with a voxel size of 1.3 μm using filtered back projection. (**c**) Samples were interrogated from the three planes. (**d**) Rendered in 3D by masking the perforations and microcracks in three planes using VGStudio MAX.

Due to the limited experiment time with the synchrotron, imaging was carried out on only 16 of the cylindrical cores (Fig. 1a). This was done with synchrotron X-ray micro-CT using Beamline I12^51^ at Diamond Light Source, United Kingdom. Six cores were from the bisphosphonate-treated group, five from the untreated fracture group and five from the healthy ageing non-fracture group. The key setting parameters used were: X-ray beam 53 KeV, image volume 23.3 mm^3^, voxel size 1.3 μm/voxel, 6400 projections, 180° rotation. Tomographic images were reconstructed using the in-house software, DAWN^52^,^53^ (Fig. 1b), which were linked to the image analysis software packages ImageJ (National Institutes of Health, Bethesda, United States of America) and VGStudio MAX (Heidelberg, Germany) for quantification of the trabecular microstructure (Fig. 1c,d).

### Mechanical Uni-Axial Tensile Experiment

Twenty-one rectangular-shaped standard tensile testing samples were obtained from the region immediately adjacent to the harvest region of the aforementioned cylindrical cores. The specimens were 11 mm in height, 2.8 mm in width, and 1 mm depth. The ends of each sample were potted in bone cement, which served as clamps and stored at −80° C till testing. During tensile testing, the samples were kept hydrated in the fluid chamber built into the micromechanical device^54^. All 21 specimens underwent load-controlled tensile testing with a fixed strain rate at 0.001 s^−1^ using a custom-built micromechanical test rig, which was designed as part of earlier work^54^. The tests were conducted at room temperature. After loading, the stress-strain curves were examined to identify the Young’s Modulus and ultimate tensile strength, which were normalised according to trabecular bone volume. Tensile testing was considered more effective for assessing microcracks.

### Microdamage Definition

**Figure 2:**
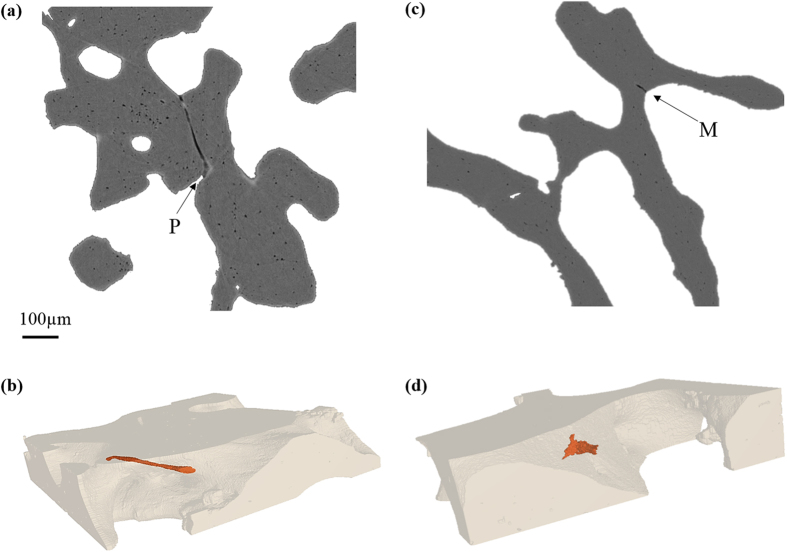
Classification of microdamage. 2D to 3D rendering of microdamage (orange) within trabecular bone (translucent). (**a,b**) Perforation (P), (**c,d**) Microcrack (M). The image analysis software, VGStudio MAX, was used to generate the 3D models using image segmentation.

The features of microdamage visualised on synchrotron X-ray micro-CT scans were classified as either perforations^27^ or microcracks^55^,^56^. Perforations^27^ are regions of complete breakage in the bone trabeculae, which are due to osteoclastic activity (Fig. 2a,b). In contrast, microcracks^55^ are microscopic fractures with a typical linear shape and sharp edges ranging between 30–100 μm in length (Fig. 2c,d).

### Microdamage Assessment

The volume analysed was 3.28 mm in diameter and 2.76 mm in height. This was done to avoid artefactual damage caused by the drilling process^57^. The region of interest was identified with an automated segmentation technique using a global threshold as described by Larrue *et al*.^18^. The scans were inspected to qualify and quantify the microdamage present in both 2D and 3D. In total, 1000 continuous slices were individually examined in three planes (Fig. 1c). Regions of microdamage were then segmented manually with localised threshold values for 3D assessment of volume as determined by the assessors who were blinded to the groups. To account for interindividual error when assessing microdamage, two independent assessors examined the CT slices (E.L.G. and B.P.). There was very high reproducibility, with an *r*^*2*^ value of 0.920 for the correlation between both assessors (Pearson’s correlation coefficient *p* = 0.010). Assessment of the scans was repeated three times by both assessors to minimise intraindividual error and data from the final assessment was used. In case of disagreement, a consensual decision between the two assessors was reached, with involvement of a third independent assessor (S.M.).

### Assessment Parameters

Trabecular bone volume fraction, microdamage density, microdamage volume, microdamage volume fraction, Young’s Modulus and ultimate tensile strength of the samples were calculated. Microdamage density and microdamage volume fraction were calculated as the frequency of microdamage and the volume of microdamage divided by the trabecular bone volume respectively. Microdamage density was used to compare the amount of microcracks and perforations across the non-fracture control, fracture control and bisphosphonate-treated groups. Meanwhile, microdamage volume was used to compare the size of microcracks and perforations across the three groups. The apparent mechanical data were divided by trabecular bone volume fraction to normalise the Young’s Modulus and ultimate tensile strength, which was to ensure a fair and valid comparison.

### Statistics

There were no relevant assumptions to construct a hypothesis for the effect size of long-term bisphosphonate treatment on microdamage accumulation in the femoral head trabecular bone cores in this study. Statistical analyses were performed using IBM SPSS Statistics 23 (Armonk, New York) and the graphs were generated with GraphPad Prism 7 (San Diego, California). The data were assessed for normality using a Q-Q plot and Shapiro-Wilk test, and were found to follow a non-Gaussian distribution. Consequently, the results were presented as median and interquartile range. Non-parametric descriptive statistics and tests used included the Kruskal-Wallis and Mann-Whitney U tests.

## Results

**Figure 3:**
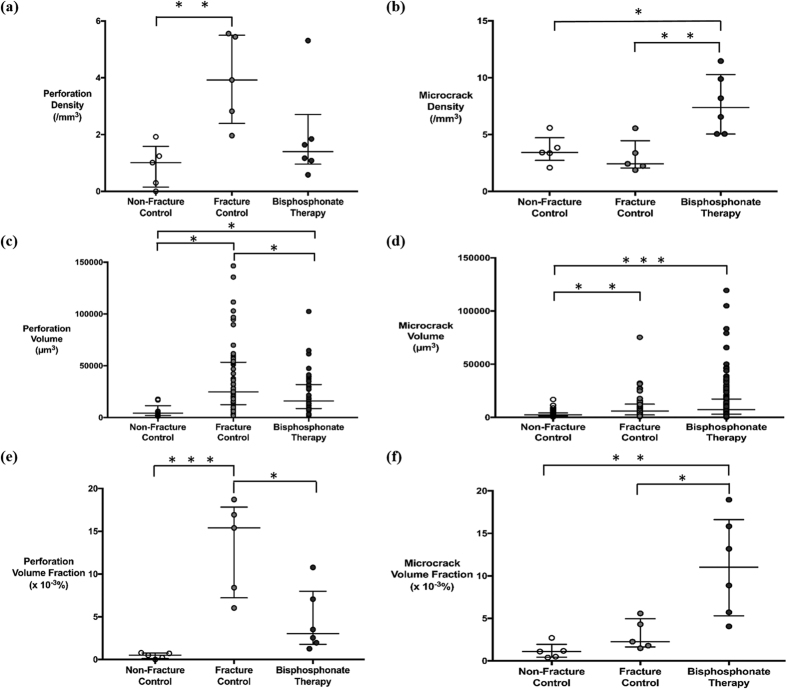
Microdamage characteristics. Median density (**a,b**), volume (**c,d**) and volume fraction (**e,f**) of perforations and microcracks in the non-fracture control (*n* = 5), fracture control (*n* = 5) and bisphosphonate therapy groups (*n* = 6). (**a**) The fracture group had the highest density of perforations at 3.92/mm^3^ across all groups but this was only significant compared to the non-fracture group (*p* = 0.005). (**b**) The bisphosphonate therapy group had a significantly higher microcrack density at 7.38/mm^3^ compared to the other two groups (fracture *p* = 0.007, non-fracture *p* = 0.012). (**c**) The fracture group had a significantly larger volume of perforations at 24863 μm^3^ compared to other two groups (bisphosphonate therapy *p* = 0.011, non-fracture *p* = 0.012). The bisphosphonate therapy group also showed a significantly higher volume of perforations at 15893 μm^3^ compared to the non-fracture group at 4220 μm^3^ (*p* = 0.019). (**d**) The bisphosphonate therapy group had the highest microcrack volume at 7173 μm^3^, which was significant compared to the non-fracture group (*p* = 0.001). The microcrack volume was also significantly lower in the non-fracture group than the fracture group (*p* = 0.004). (**e)** The fracture group had the greatest perforation volume fraction at 15.39 × 10^−3^%, which was significantly greater than both the bisphosphonate-treated group, which was 3.02 × 10^−3^% and non-fracture group, which was 0.50 × 10^−3^% (bisphosphonate therapy *p* = 0.013, non-fracture *p* = 0.001). (**f**) The bisphosphonate therapy group had the highest microcrack volume fraction at 11.03 × 10^−3 ^% (fracture *p* = 0.017, non-fracture *p* = 0.005). Statistically, microdamage data were compared using a Kruskal-Wallis and Mann-Whitney U test. Asterisks denote significant pairwise differences at *p < 0.050, **p < 0.010 and ***p < 0.001.

The fracture control group had the highest density of perforations across all groups (*p* = 0.040). This was significantly higher than the non-fracture group but was not significant compared to the bisphosphonate-treated group (Fig. 3a). Similarly, the fracture control group displayed the largest volume of perforations, followed by the bisphosphonate-treated and the non-fracture control group (*p* = 0.030). These differences were all statistically significant (Fig. 3c). The fracture group also had the highest perforation volume fraction, which was statistically significant compared to the other two groups *(p* = 0.0150) (Fig. 3e).

The bisphosphonate-treated group had the highest density of microcracks compared to the fracture and non-fracture control groups, which was statistically significant (*p* = 0.010) (Fig. 3b). Both the fracture and non-fracture control groups had a comparable density of microcracks. Moreover, the bisphosphonate-treated group had the largest volume of microcracks, followed by the fracture and non-fracture control groups (*p* = 0.001). This difference was significant compared to the non-fracture control group but not the fracture control group. The volume of microcracks in the bisphosphonate-treated group was also significantly greater compared to the non-fracture control group (*p* = 0.004) (Fig. 3d). Meanwhile, the bisphosphonate-treated group had the highest microcrack volume fraction compared to the fracture and non-fracture groups (*p* = 0.020) (Fig. 3f).

**Figure 4:**
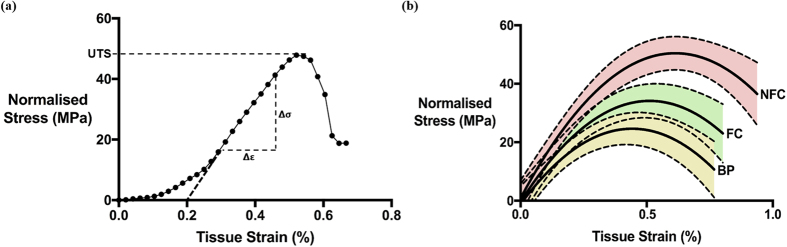
Tensile stress-strain curves. (**a**) An example of a stress-strain curve. The Young’s Modulus was obtained by calculating the gradient of the linear section of the curve. The ultimate tensile strength was obtained from the maximum stress value of the curve. (**b**) Overall stress-strain curves are shown for the non-fracture control (NFC), fracture control (FC) and bisphosphonate-treated (BP) groups. The shaded area represents the 95% confidence intervals for each group.

**Figure 5:**
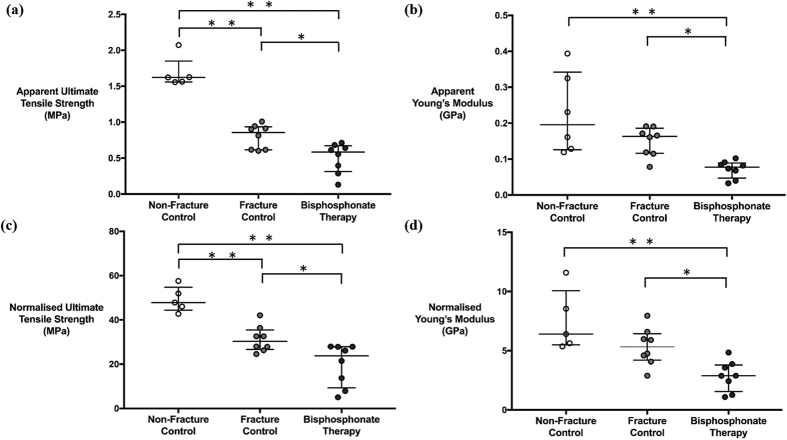
Mechanical data characteristics. Median apparent and normalised ultimate tensile strength and Young’s Modulus in the non-fracture control, fracture control and bisphosphonate therapy groups. (**a**) The bisphosphonate therapy group had a significantly lower apparent ultimate tensile strength at 0.58 MPa compared to the other two groups (fracture *p* = 0.021, non-fracture *p* = 0.002). Non-fracture group also had a significantly higher apparent ultimate tensile strength at 1.62 MPa than fracture group at 0.86 MPa (p = 0.002). (**b**) The bisphosphonate therapy group showed a significantly lower apparent Young’s Modulus at 0.070 GPa than the fracture group at 0.16 GPa and non-fracture group at 0.20 GPa (fracture *p* = 0.021, non-fracture *p* = 0.002). (**c**) The bisphosphonate therapy group had a significantly lower normalised ultimate tensile strength at 23.78 MPa compared to the other two groups (fracture *p* = 0.028, non-fracture *p* = 0.002). Non-fracture group also had a significantly lower ultimate tensile strength at 30.28 MPa than fracture group at 47.86 MPa (*p* = 0.002). (**d**) The bisphosphonate therapy group showed a significantly lower normalised Young’s Modulus at 2.88 GPa than the fracture group at 5.33 GPa and non-fracture group at 6.41 GPa (fracture *p* = 0.028, non-fracture *p* = 0.002). The mechanical data were compared using a Kruskal-Wallis and Mann-Whitney U test. Asterisks denote significant pairwise difference at *p < 0.050, **p < 0.010, ***p < 0.001.

The trabecular bone volume fraction in the healthy aging non-fracture group was 0.344 ± 0.030. This was significantly higher than the untreated fracture group at 0.241 ± 0.025 (*p* = 0.001) but not compared to the bisphosphonate-treated fracture group at 0.288 ± 0.069. The overall stress-strain curves with the 95% confidence intervals across the three groups were generated (Fig. 4). Data for the apparent (Fig. 5a,b) and normalised (Fig. 5c,d) ultimate tensile strength and Young’s Modulus displayed a similar trend. The bone volume normalised ultimate tensile strength was the highest in the non-fracture group, followed by the fracture and bisphosphonate-treated groups. Statistically significant differences were present between all the groups (*p* = 0.001) (Fig. 5c). Additionally, the bisphosphonate therapy group showed a significantly lower normalised Young’s Modulus compared to the fracture and non-fracture groups (*p* = 0.030) (Fig. 5d).

## Discussion

This is the first study to investigate and compare the morphology of microdamage in healthy, osteoporotic and bisphosphonate-treated bone using synchrotron X-ray micro-CT and image segmentation technology. Trabecular microdamage and mechanical strength were compared across the three groups. Bone from the fracture control group exhibited the highest density of perforations across the three groups. Meanwhile, bone from the bisphosphonate-treated group demonstrated the highest density of microcracks. Microcracks were typically larger in bone from the bisphosphonate-treated group, while perforations were larger in bone from the fracture control group. Accordingly, the bisphosphonate-treated and fracture control groups had the highest microcrack and perforation volume fractions, respectively. Bone from the non-fracture control group had the highest normalised Young’s Modulus and tensile strength, followed by bone from the fracture control and bisphosphonate-treated groups.

A limitation of the study was the small sample size: 16 trabecular bone cores and 21 rectangular tensile samples were investigated. In addition, synchrotron X-ray micro-CT has the potential to cause radiation damage, which may lead to artefacts in the scans. To account for this, the energy settings were calibrated to be lower than 30–35 kGy (safe level) and the exposure time of the scan was kept under 30 seconds per frame^58^. Another limitation of the study was that the bones cores in the bisphosphonate-treated and fracture-control groups were harvested from femoral heads following hip fracture, which should be kept in mind when generalising the results. To do so, further studies of bone from patients treated with bisphosphonates without fracturing should be performed. Finally, the duration of bisphosphonate treatment was not controlled in this study. However, all samples were obtained from patients receiving treatment for a minimum of one year^59^.

In the present study, bone from the fracture control group exhibited the highest density and volume of perforations across all groups. Bone from healthy ageing individuals demonstrated a comparable density of microcracks with bone from fracture controls, although the volume of microcracks was significantly larger in the latter group. In healthy bone, sustained loading and fatigue caused by normal physical activity leads to microdamage formation, which results in a loss of mechanical integrity^47^. In order to maintain the mechanical integrity of bone, the remodelling process is targeted at sites of microdamage to initiate repair^55^,^60^. In osteoporosis, osteoclast activity is markedly elevated, which explains the high density and volume of perforations observed in this study^61^. Thus, the reduction in the normalised Young’s Modulus and ultimate tensile strength of untreated fracture bone compared to healthy bone can be attributed to a loss of structural integrity caused by perforations. Given that more than half of patients suffering from fragility fractures do not fit a DXA-based clinical diagnosis of osteoporosis^15^,^16^, these findings suggest that osteoporosis may not solely be a disorder characterised by loss of bone mass, but also the disruption of bone microstructure^17^ due to perforations.

Bisphosphonate-treated bone from fracture patients had the highest density and volume of microcracks compared to bone from the untreated fracture patients and healthy ageing individuals. Correspondingly, bisphosphonate-treated samples also had reduced ultimate tensile strength and Young’s Modulus compared to the control groups. Our results, therefore, suggest that the reduced bone strength in the bisphosphonate group is due to the accumulation of microcracks. In this subgroup of bisphosphonate-treated patients that suffered a fracture, the accumulation of microcracks following treatment with bisphosphonates may have compromised the trabecular microstructure. As a result, there may have been weakening of the bone and consequently, an increased risk of fracture. Bisphosphonate-treated bone also demonstrated a lower density and volume of perforations compared to osteoporotic bone, which may be reflective of the protective effects of bisphosphonates in limiting the development of perforations through osteoclastic inhibition^27^. However, it is the oversuppression of remodelling that has detrimental effects, as this predisposes to microcrack accumulation and propagation^43^.

Bone from healthy ageing individuals demonstrated a comparable density of microcracks with bone from fracture patients although the volume was substantially lower. The accumulation of microcracks can potentially weaken bone and reduce tensile strength but microcracks are a part of normal physiological toughening mechanisms^2^,^49^,^62^. Bone is a hierarchical structure with energy-dissipating mechanisms at each level^63^. At the microstructural level, the formation of small microcracks can be an effective strategy for dissipating energy. Healthy bone has mechanisms to sustain microcrack accumulation, by limiting propagation and inducing an appropriate remodelling response to initiate repair. The toughness of bone is therefore maintained through the balance between the protective effects conferred by the fracture toughening mechanisms in bone and the formation of microcracks^64^. Disruption of this balance leads to a loss of heterogeneity of the trabecular composition, which is strongly correlated with a reduction in fracture toughness^35^,^65^,^66^. Thus, the dysregulation of the remodelling process that occurs in osteoporosis and with bisphosphonate therapy predisposes to the formation and propagation of microcracks, which may occur through cracking of bone mineral crystallites, debonding at the mineral organic interface or shear between and within collagen fibrils^67^.

Consistent with the findings of the present study, Zimmermann *et al*. noted that cortical bone samples from osteoporotic and bisphosphonate-treated individuals displayed reduced bending modulus, yield stress and maximum bending stress compared to healthy adults^6^. Similarly, Lambers *et al*. reported an inverse relationship between bone microdamage volume fraction and Young’s Modulus caused by fatigue loading. Additionally, the authors observed that samples with microdamage were able to withstand 92% fewer cycles before failure compared to the same tissue without microdamage in their study^49^. These findings suggest that even small quantities of microdamage can have a significant effect on the biomechanical performance of bone. Using nanoindentation techniques, Wang *et al*. noted a reduction in bone stiffness and elasticity following the formation of microdamage, which was maintained even after repair had occurred^68^. Our findings corroborate and build on previous analyses by establishing the differences in microdamage in healthy ageing, diseased and treated bone and correlating these with the mechanical properties.

In humans, there is limited evidence regarding the effects of long-term (>5 years) bisphosphonate therapy and microcrack accumulation. Clinical trials report a reduction in fracture risk but data available is limited to the duration of the trials, which typically last between 3–4 years^29^,^30^,^69^,^70^. This is very important clinically, as patients with osteoporosis are usually placed on treatment lasting more than five years. A recent nested case-control study by Erviti *et al*., who followed a cohort of 12054 individuals, reported patients on bisphosphonate therapy for longer than 3 years to be at significantly elevated risk of hip fracture compared to the control group. Furthermore, fracture risk was found to be independently associated with the duration of treatment^71^. Thus, the occurrence of hip fractures in bisphosphonate-treated patients may not be due to the inefficacy of the drug but rather a consequence of its effectiveness. Pooled analysis of three long-term extension trials on alendronate, risedronate and zoledronate revealed marginally lower hip fracture rates in patients switching to the placebo compared to those persisting with treatment^72^. The BMD in the femoral neck of patients who persisted with treatment was maintained but declined in those that had switched to the placebo. Given these findings, it is unlikely that these differences in risk can be reliably attributed to BMD. The increased incidence of fractures noted in these studies may be due to the accumulation of microcracks in the femoral neck, which can impair the mechanical properties of the bone, thereby predisposing to fracture. Moving forwards, our findings will form the groundwork for studies investigating the structure of bone at a micro- and nanostructural level, which will provide us with a more complete picture.

## Conclusion

In this study, bone from bisphosphonate-treated fracture patients exhibited fewer perforated trabeculae compared to bone from untreated fracture patients, which suggests successful suppression of the remodelling process. However, more microcracks were present, which were larger in volume compared to untreated fracture and healthy ageing non-fracture controls. Given the reduced mechanical strength noted in the bone samples from the bisphosphonate-treated group, these microstructural changes may explain the mechanism by which fractures occur in patients treated with bisphosphonates. Thus, it is plausible that there may be a population of patients in whom bisphosphonate therapy does not confer protective effects in resisting fractures, but is associated with microstructural damage and increased bone fragility instead. For patients on long-term therapy, if this microcrack accumulation critical time-point could be predicted, bisphosphonate treatment duration could be optimised. Bisphosphonates could be prescribed for long enough to increase bone volume and reduce perforations, whilst stopping the medication before a critical accumulation of microcracks. Whilst it may be premature to rethink current fracture prevention strategy, caution should be exercised when prescribing bisphosphonates to all patients deemed at risk of fracture and the duration of treatment should be carefully considered.

## Additional Information

**How to cite this article**: Ma, S. *et al*. Long-term effects of bisphosphonate therapy: perforations, microcracks and mechanical properties. *Sci. Rep.* **7**, 43399; doi: 10.1038/srep43399 (2017).

**Publisher's note:** Springer Nature remains neutral with regard to jurisdictional claims in published maps and institutional affiliations.

## Figures and Tables

**Table 1:** Demographics for bisphosphonate therapy group.

Sample	Sex	Age	Bisphosphonate	Dosage (mg/week)	Treatment Period (year)	X-ray Micro-CT Scan	Tensile Testing
BP-1	F	88	Alendronic Acid	70	9	Yes	Yes
BP-2	F	61	Alendronic Acid	70	5.5	Yes	Yes
BP-3	F	79	Alendronic Acid	70	5	Yes	Yes
BP-4	F	82	Alendronic Acid	70	5	Yes	Yes
BP-5	F	68	Alendronic Acid	70	1	Yes	Yes
BP-6	M	80	Alendronic Acid	70	1	Yes	Yes
BP-7	F	84	Alendronic Acid	70	5	No	Yes
BP-8	F	82	Alendronic Acid	70	2	No	Yes
